# 基于配体垂钓技术筛选三七叶中环氧合酶-2抑制剂

**DOI:** 10.3724/SP.J.1123.2024.07003

**Published:** 2024-12-08

**Authors:** Fan ZHANG, Wei WANG, Ying CAO, Yi ZHANG, Lijie WU

**Affiliations:** 天津中医药大学中药学院, 天津 301617; College of Chinese Materia Medica, Tianjin University of Traditional Chinese Medicine, Tianjin 301617, China

**Keywords:** 配体垂钓, 环氧化酶-2抑制剂, 三七叶, 皂苷, 抗炎, ligand fishing, cyclooxygenase-2 inhibitor (COX-2 inhibitor), *Panax notoginseng* leaves, saponins, anti-inflammatory

## Abstract

市售环氧合酶-2(COX-2)抑制剂如美洛昔康、塞来昔布等与传统的非甾体抗炎药相比虽能够有效治疗炎症,但是也存在着引起肝毒性、心血管疾病的风险。中药三七、人参等被证实具有良好的抗炎作用且毒副作用小,因此从中药中筛选潜在的COX-2抑制剂对于开发安全有效的新型抗炎药具有重要意义。本研究以磁性纳米材料作为高效载体,采用配体垂钓法替代传统低效筛选手段,结合固定化金属亲和技术,解决了传统酶固定方法带来的酶活性降低或酶分子堆积等问题。首先合成了聚多巴胺(PDA)包覆的Fe_3_O_4_,在PDA表面螯合Ni^2+^,利用金属离子亲和作用固定COX-2,制备了一种绿色环保、特异性好的垂钓工具。采用扫描电子显微镜、透射电子显微镜、X射线光电子能谱仪、热重分析仪、振动样品磁强计等方法对其结构进行了表征,结果显示磁性纳米材料为核壳结构,粒径为250~300 nm,具有较大的比表面积,且磁性强、稳定性好。以COX-2抑制剂塞来昔布为垂钓目标物对COX-2的固定条件及垂钓条件进行了优化。该法成功应用于三七叶中潜在COX-2抑制剂的筛选,最终得到了13种能与COX-2相互作用的成分,经液相色谱-质谱联用技术鉴定均为20(*S*)-原人参二醇型皂苷。与传统的筛选方法相比,本方法简便快速,酶活性高,能够实现对特定目标物的捕获与富集。该研究对新型抗炎药的合成与开发具有指导意义,为高效地从中药复杂体系中发现抗炎药物或先导化合物提供了借鉴,同时也为三七叶的资源化合理应用提供了新的思路。

## Abstract (en)

Human health is increasingly affected by chronic inflammation, which can subsequently develop into chronic diseases such as cancer, arthritis, diabetes, and diseases of the cardiovascular and nervous systems. Traditional non-steroidal anti-inflammatory drugs (NSAIDs) that inhibit cyclooxygenase-1 (COX-1) produce side effects, such as irritation of the gastrointestinal tract and renal toxicity. While commercially available inhibitors of the cyclooxygenase-2 (COX-2) enzyme, such as meloxicam and celecoxib, treat inflammation more-effectively than traditional NSAIDs, they associate with liver-toxicity and cardiovascular-disease risks. Modern pharmacological studies have shown that *Panax ginseng*, *Panax notoginseng*, and other traditional Chinese medicines have anti-inflammatory properties; these medicines mainly contain flavonoids, alkaloids, saponins, polysaccharides, and other anti-inflammatory components, are highly efficient, and exhibit few side effects, which is advantageous. Therefore, screening potential COX-2 inhibitors from traditional Chinese medicine is greatly significant for the development of safe and effective novel anti-inflammatory drugs. However, screening these traditional medicines in an easy, quick, accurate, and efficient manner presents problem that require urgent solutions because traditional screening methods are cumbersome and inefficient.

In this study, we used the ligand fishing method instead of poorly efficient traditional screening methods, with magnetic nanoparticles used as the carrier owing to their advantageous fast separation speeds. In addition, we also maintained the spatial structure and activity of the fixed enzyme by combining immobilized metal affinity technology, and selected polydopamine (PDA) as a low-toxicity metal-chelating agent owing to its strongly adhesive nature and good chelating ability for various metal ions. The application of PDA solves the problems of metal-ion leakage and limited protein load associated with traditional chelating agents. Firstly, magnetic Fe_3_O_4_ nanoparticles were hydrothermally synthesized, after which their surfaces were coated with PDA using the magnetic-stirring method, with Ni^2+^ then chelated on the PDA surface and COX-2 fixed through metal-ion affinity, to afford a convenient, fast, green, and specific fishing tool. The structure and properties of the magnetic nanomaterial were characterized by scanning electron microscopy (SEM), transmission electron microscopy (TEM), X-ray photoelectron spectroscopy (XPS), thermogravimetric analysis, vibrating sample magnetometry (VSM), and other methods. The magnetic nanomaterials have core-shell structures with particles that are 250-300 nm in size; they also have a large specific surface area, are strongly magnetic, highly stable, and perform excellently. Celecoxib, a selective COX-2 inhibitor, was used as a fishing target during optimization of the fixed-COX-2 and fishing conditions, including the adsorption and elution conditions. The optimal settings were then successfully used to screen potential COX-2 inhibitors in *Panax notoginseng* leaves. Methodological experiments revealed that the instrument and method are highly precise, accurate, and repeatable, with a limit of detection (LOD) of 0.02 mg/mL and a limit of quantification (LOQ) of 0.07 mg/mL. Only celecoxib was detected in a fishing solution composed of glipizide, indomethacin, and celecoxib, which implies that immobilized COX-2 exhibits good fishing specificity, and that only components that strongly interact with COX-2 are caught. Thirteen components that interact with COX-2 were identified by liquid chromatography-mass spectrometry for a fishing solution of *Panax notoginseng* leaves, including notoginsenoside Fa and ginsenoside Rb_1_, which are expected to be COX-2 inhibitors. Compared with traditional screening methods, the method developed in this study is simple, rapid, and highly specific and enzyme active, leading to the capture and enrichment of the specific target. This study provides guidance for the synthesis and development of new safe and effective anti-inflammatory drugs, and provides a reference for the efficient discovery of such drugs or lead compounds from complex traditional Chinese medicines; it also provides a new concept for the rational utilization of *Panax notoginseng* leaves.

慢性炎症已逐渐成为威胁人类健康的疾病之一,慢性炎症极易发展成为癌症、关节炎、糖尿病、心血管疾病以及神经系统疾病等慢性疾病^[1,2]^。已有研究发现存在两种与之密切相关的酶即环氧合酶-1(COX-1)和环氧合酶-2(COX-2),其中COX-1与生理性前列腺素的合成有关,维持机体正常生理活动,而COX-2则是诱导型酶,响应炎症表达,诱导炎症因子的产生,促使炎症加剧^[3]^。非甾体抗炎药(NSAIDs)是目前常用的抗炎药物,主要通过抑制环氧合酶(COX)的活性,进而阻断前列腺素的合成及炎症因子的产生等,从而发挥抗炎作用^[4]^。传统的NSAIDs在抑制COX-1的过程中,往往会导致胃肠道不适及肾脏毒性等副作用的产生^[5]^,而选择性COX-2抑制剂由于副作用小的特点受到广泛的青睐,如罗非昔布、美洛昔康及塞来昔布等,但是随着研究深入发现,选择性COX-2抑制剂也存在着引起肝毒性、心血管风险的问题^[6,7]^,因此寻找和开发安全有效的新型COX-2抑制剂具有重要的意义。现代药理学研究发现中药如金银花、人参、三七、钩藤等具有抗炎功效,主要含有黄酮、生物碱、皂苷、多糖等抗炎药效成分,具有高效、副作用小等优点^[8,9]^。因此从中药中筛选潜在的COX-2抑制剂已成为备受关注的热点,然而如何简便快速且准确高效地筛选是目前亟待解决的问题。

磁性配体垂钓是以磁性纳米粒子(MNPs)作为载体,通过交联、包埋、吸附或共价连接等方法将酶固定在MNPs上,利用配体-受体作用将目标成分垂钓出来^[10]^;但是上述酶固定方法会产生酶固定后活性降低或酶分子堆积的问题,影响垂钓的灵敏度与效率^[11]^。固定化金属离子亲和技术(IMAN)常用于吸附分离纯化多肽或蛋白,能够有效保持蛋白的空间结构与活性。但IMAN常用的螯合剂与偶联剂如亚氨基二乙酸、亚硝基三乙酸等存在金属离子易泄露、蛋白载量有限的问题^[12]^,且部分偶联剂价格昂贵、毒性大,导致实验成本及危险性大大增加。聚多巴胺(PDA)由多巴胺在弱碱条件下聚合而成,具有较强的黏附与交联能力,其丰富的邻苯二酚结构对多种金属离子如Ti^2+^、Ni^2+^、Zn^2+^、Fe^3+^等具有较强的螯合能力,是一种理想的金属螯合剂^[13]^。

三七叶是植物三七的干燥叶,主要含有三萜皂苷类、黄酮类、多糖等成分,具有抗炎、抗氧化、降血糖等作用^[14,15]^。目前已有研究报道三七叶中几乎只含有20(*S*)-原人参二醇型皂苷,而三七根部含有较多的20(*S*)-原人参三醇型皂苷^[16,17]^。成分的差异预示着三七叶除具有与根部相同的药理作用外还具有独特的作用, 有待深入研究,为潜在的COX-2抑制剂的筛选提供了新的思路。

本研究将磁性配体垂钓技术与固定化金属离子亲和技术结合,选用PDA代替传统的螯合剂,包覆在Fe_3_O_4_表面,并与Ni^2+^络合,依靠Ni^2+^的亲和作用固定COX-2用于三七叶中皂苷类成分的垂钓,以筛选潜在的COX-2抑制剂成分,为新型抗炎药物的开发提供借鉴。

## 1 实验部分

### 1.1 仪器、试剂与材料

Acquity UPLC/Xevo G2-XS QTof超高效液相色谱-四极杆飞行时间质谱仪(美国Waters公司,用于实际样品测定); Alliance 2695型高效液相色谱仪(美国Waters公司,用于条件优化实验); SYWF-50水浴恒温振荡器(天津市莱玻特瑞仪器设备有限公司); FEI Scios 2扫描电子显微镜(SEM,美国Thermo公司); FEI Tecnai F20透射电子显微镜(TEM,美国FEI公司); Thermo Scientific ESCALAB 250Xi X射线光电子能谱仪(XPS,美国Thermo公司); Micromeritics ASAP 2460全自动比表面及孔隙度分析仪(BET,美国Micromeritics公司); LakeShore 7404振动样品磁强计(VSM,美国LakeShore公司); TGA 550热重仪(美国Discovery公司); DGG-9140A电热恒温鼓风干燥箱(上海培因实验仪器有限公司); DW-1磁力恒速搅拌器(巩义市予华仪器有限责任公司); DZG-6050真空干燥箱(上海培因实验仪器有限公司)。

六水合三氯化铁(FeCl_3_·6H_2_O, AR, 99%)、聚乙二醇6000(*M*_n_ 6000)、三羟甲基氨基甲烷(Tris, ≥99.8%)、盐酸多巴胺(DA, 98%)均购自上海阿拉丁生化科技股份有限公司;无水乙酸钠(≥99.0%)购自天津为科生物有限公司;Tris-HCl缓冲溶液(0.01 mol/L, pH 8.0)购自上海源叶生物科技有限公司;六水合氯化镍(NiCl_2_·6H_2_O,分析纯)购自天津渤化化学试剂有限公司;乙二醇(分析纯,99.5%)、无水乙醇(分析纯,99.7%)、甲醇(色谱纯,99.9%)、乙腈(色谱级,99.9%)购自天津康科德有限公司;塞来昔布标准品(≥98%)、格列吡嗪标准品(≥98%)、吲哚美辛标准品(≥98%)和二甲基亚砜(DMSO,ACS级,≥99.9%)购自北京索莱宝科技有限公司;COX-2(>90%)购自武汉爱博泰克生物科技有限公司。

实验所用皂苷标准品来源于实验室前期三七叶活性成分分离鉴定实验^[18]^。

### 1.2 固定化COX-2的制备

#### 1.2.1 Fe_3_O_4_的制备

首先称取2.7 g FeCl_3_·6H_2_O,加入80 mL乙二醇搅拌至溶解,再加入7.2 g无水乙酸钠和2 g聚乙二醇6000,于室温下搅拌30 min,待搅拌均匀后转移至具聚四氟乙烯内衬的反应釜中200 ℃下反应8 h。反应结束后,静置冷却至室温,利用磁铁分离得到黑色固体颗粒,并依次用超纯水与无水乙醇洗涤数次,于50 ℃真空干燥箱中干燥过夜即得。

#### 1.2.2 Fe_3_O_4_@PDA的制备

称取400 mg制备得到的Fe_3_O_4_分散于250 mL Tris-HCl溶液(5 mg/mL, pH=8.5, 自制)中,并加入500 mg盐酸多巴胺,室温下搅拌10 h。搅拌结束后通过磁铁收集固体颗粒并用超纯水和无水乙醇洗涤,于50 ℃真空干燥箱中干燥即得。

#### 1.2.3 Fe_3_O_4_@PDA-Ni^2+^的制备

称取100 mg Fe_3_O_4_@PDA分散于100 mL NiCl_2_ (2.5 mg/mL)溶液中,室温下搅拌12 h,然后磁分离得到固体颗粒并用超纯水和无水乙醇洗涤,于50 ℃真空干燥箱中干燥过夜即得。

#### 1.2.4 COX-2的固定

称取0.4 mg Fe_3_O_4_@PDA-Ni^2+^,加入300 μL Tris-HCl缓冲溶液(0.01 mol/L, pH 8.0,下文无特殊标注均为此浓度和pH值)平衡1 h,由磁分离除去上清液,然后加入800 μL含COX-2的Tris-HCl缓冲溶液(COX-2质量浓度为0.16 μg/mL),于4 ℃下振荡2 h。通过磁分离收集并用超纯水洗涤,然后分散于100 μL Tris-HCl缓冲溶液中4 ℃保存,即为固定COX-2的MNPs缓冲溶液(4 mg/mL)。

### 1.3 实际样品垂钓

#### 1.3.1 三七叶样品溶液的制备

称取干燥的三七叶1 kg,剪碎,加入10 L 50%乙醇水溶液加热回流提取3次(3、2、2 h),合并3次提取液,减压回收溶剂得到浓缩的三七叶粗提液。粗提液经大孔树脂D101吸附12 h后,依次用水、30%乙醇水溶液、80%乙醇水溶液进行洗脱。收集80%乙醇水溶液洗脱液,减压干燥即得三七叶总皂苷粉末。称取500 mg总皂苷粉末,加入25 mL甲醇溶解,即为三七叶样品溶液。

#### 1.3.2 样品垂钓

将50 μL Tris-HCl缓冲溶液及9 μL三七叶样品溶液加入到100 μL含有固定COX-2的MNPs缓冲溶液(4 mg/mL)中,35 ℃下振荡1 h后磁分离收集MNPs,并用缓冲溶液洗涤数次以除去未反应的成分。然后加入400 μL甲醇孵育10 min,磁分离得到洗脱液,通过超高效液相色谱-四极杆飞行时间质谱(UPLC-Q-TOF-MS)分析鉴定成分结构。

### 1.4 色谱及质谱条件

#### 1.4.1 条件优化及方法学考察色谱条件

色谱柱:Symmetry C18(250 mm×4.6 mm, 5 μm);流动相:0~8 min,水-甲醇(15∶85,v/v);柱温为25 ℃;流速为1 mL/min;进样量为10 μL;检测波长为254 nm。

#### 1.4.2 特异性垂钓验证色谱条件

色谱柱为Symmetry C18(250 mm×4.6 mm, 5 μm);流动相:A为0.1%甲酸水溶液,B为乙腈;洗脱梯度:0~40 min,5%B~95%B;流速:0.7 mL/min;柱温:25 ℃;进样量:10 μL;检测波长:258 nm。

#### 1.4.3 UPLC-Q-TOF-MS条件

色谱条件 色谱柱:ACQUITY UPLC BEH C18(100 mm×2.1 mm, 1.7 μm);流动相:A为0.1%甲酸水溶液,B为乙腈。洗脱梯度:0~1.5 min, 10%B; 1.5~3 min, 10%B~15%B; 3~10 min, 15%B~25%B; 10~35 min, 25%B~40%B; 35~40 min, 40%B~70%B; 40~42 min; 70%B~100%B; 42~43.5 min, 100%B; 43.5~44 min, 100%B~10%B; 44~46 min, 10%B。流速:0.3 mL/min;进样量:5 μL;柱温:35 ℃;检测波长:254 nm。

质谱条件 ESI离子源,负离子模式检测,扫描范围:*m/z* 100~2000;毛细管电压:3000 V;孔锥电压:30 V;离子源温度:120 ℃;脱溶剂管温度:350 ℃;雾化气流速:50 L/h;脱溶剂气(N_2_)流速:500 L/h;采集时间:0~46 min。

## 2 结果与讨论

### 2.1 结构表征

#### 2.1.1 SEM及TEM表征

SEM及TEM表征结果如图1所示,可以看到合成的MNPs粒径为250~300 nm,呈均匀的球形,表面凹凸不平,具有较多的孔隙;且在TEM图(图1b)中可以较为明显地看到MNPs具有核壳结构,表明PDA已成功包覆。

**图 1:**
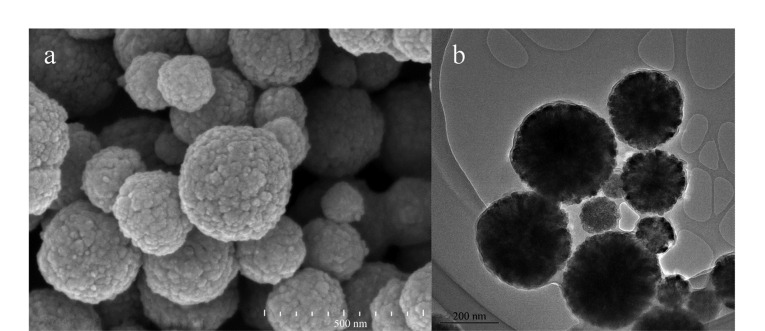
Fe_3_O_4_@PDA-Ni^2+^的(a)SEM及(b)TEM表征图

#### 2.1.2 XPS分析

通过XPS对MNPs中的元素成分、价态及比例进行分析,测得宽谱与窄谱扫描结果如图2所示,得到了C 1*s*、N 1*s*、O 1*s*、Fe 2*p*、Ni 2*p*的特征峰,并对各元素进行定量,见表1。各元素价态及比例信息表明MNPs中确实存在Fe_3_O_4_、PDA、Ni^2+^,即材料成功合成。

**图 2:**
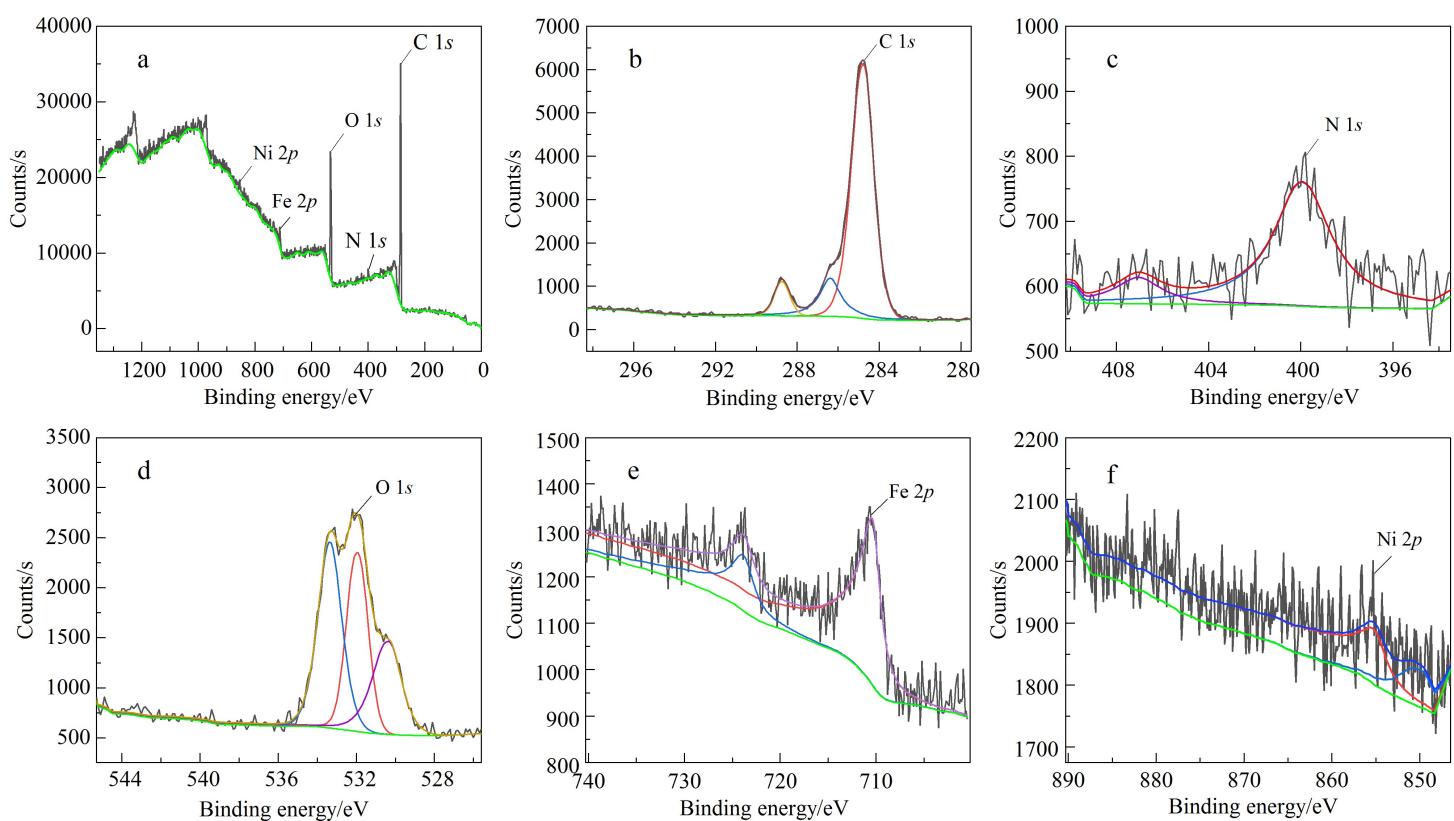
(a)Fe_3_O_4_@PDA-Ni^2+^的X射线光电子能谱宽谱及(b-f)C 1*s*、N 1*s*、O 1*s*、Fe 2*p*、Ni 2*p*窄谱扫描图

**表 1:** C 1*s*、O 1*s*、N 1*s*、Fe 2*p*、Ni 2*p*的峰位及原子比

Name	Binding energy/eV	Atomic/%
C 1s	284.94	43.46
O 1s	532.36	11.17
N 1s	399.81	0.92
Fe 2p	710.96	0.46
Ni 2p	852.95	0.41

#### 2.1.3 热重分析

如图3a所示,随着温度由室温升至800 ℃, MNPs逐渐分解,直至800 ℃时质量趋于平稳,质量损失约8%。质量变化约分为3个阶段,第一阶段为室温~200 ℃, MNPs质量有较小减少,此阶段主要为水分的蒸发;200~300 ℃阶段,MNPs质量有明显减少,推测该阶段为表面的Ni^2+^与PDA之间的作用键断裂;300~800 ℃阶段,MNPs质量逐步减少并在800 ℃左右趋于平稳,在744 ℃左右质量变化较快,表明此时MNPs中的PDA层急剧分解,最终剩下Fe_3_O_4_不再分解,质量不再变化。

**图 3:**
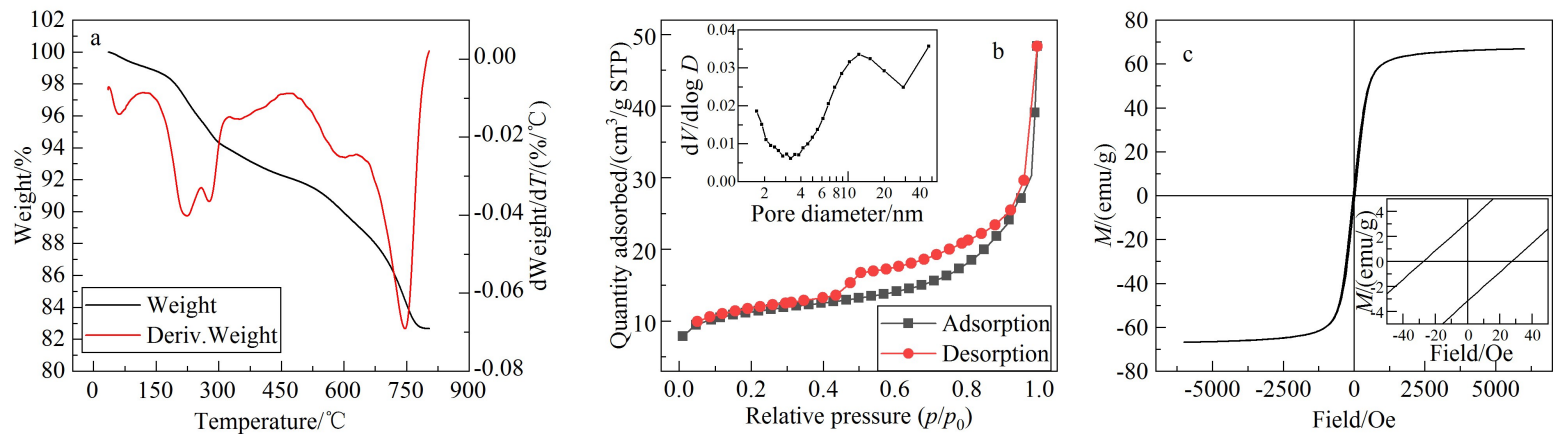
Fe_3_O_4_@PDA-Ni^2+^的(a)热重分析、(b)比表面及孔隙度分析及(c)磁性能表征结果

#### 2.1.4 BET分析

MNPs的氮气吸/脱附等温曲线如图3b所示,为标准的Ⅱ型吸附等温线及H4型回滞环等温线,其吸附饱和平台不明显,表明MNPs表面孔结构不规整。由BET模型计算得到MNPs的比表面积为41.1886 m^2^/g。由孔径分布图可以看到,MNPs表面多分布介孔,其平均孔径为11.7361 nm。

#### 2.1.5 VSM分析

采用振动样品磁强计对MNPs的磁性能进行表征,其磁滞回线见图3c,呈S形,饱和磁化强度为66.83 Oe,表明材料在包覆PDA后仍然有较强的磁性,且由磁滞回线的局部放大图可知,MNPs的矫顽力约为25 Oe,表明材料磁性能较稳定且具有较强的顺磁性。

### 2.2 COX-2固定条件优化

实验选用COX-2特异性抑制剂塞来昔布作为垂钓目标物,用于垂钓条件的优化。以高效液相色谱(HPLC)测定洗脱液中塞来昔布的质量浓度,由测得垂钓前后塞来昔布的质量浓度计算垂钓效率,计算公式如下:垂钓效率=*CV/C*_0_*V*_0_×100%,其中*C*为洗脱液中塞来昔布的质量浓度,mg/mL; *V*为洗脱液体积,mL; *C*_0_为垂钓前塞来昔布溶液的质量浓度,mg/mL; *V*_0_为垂钓前塞来昔布溶液的体积,mL。

#### 2.2.1 Ni^2+^质量浓度优化

考察了不同Ni^2+^质量浓度对配体垂钓效果的影响,选择最优的Ni^2+^质量浓度以最大效率固定COX-2。分别设置了1、1.5、2、2.5、3 mg/mL的NiCl_2_,按1.2.4节方法分别固定COX-2。然后分别加入50 μL Tris-HCl缓冲溶液与9 μL塞来昔布溶液(5 mg/mL, DMSO),按1.3.2节条件进行吸附与洗脱,将得到的洗脱液分别进行HPLC分析,计算垂钓效率。结果如图4a所示,随着Ni^2+^质量浓度的增加,固定化COX-2的垂钓效率逐渐升高,在质量浓度为2.5 mg/mL时最高,而当质量浓度变为3 mg/mL时,垂钓效率急剧降低,推测可能Ni^2+^浓度过高会破坏MNPs表面的PDA层,导致酶固定量减少,故选择质量浓度为2.5 mg/mL的Ni^2+^合成MNPs。

**图 4:**
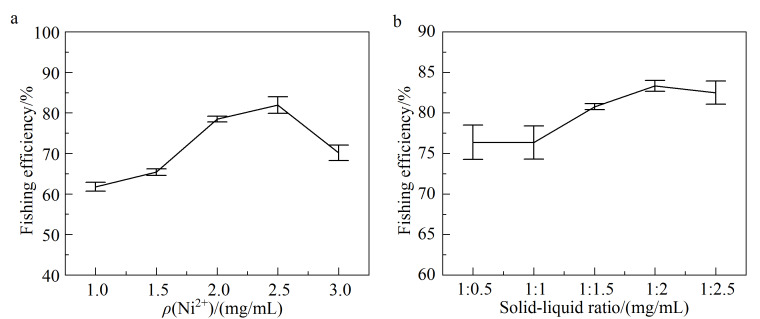
固定化COX-2在不同(a)Ni^2+^质量浓度及(b)料液比条件下的垂钓效率(*n*=3)

#### 2.2.2 料液比优化

选择了不同料液比(即MNPs的质量与COX-2溶液体积比(mg∶mL), 1∶0.5、1∶1、1∶1.5、1∶2、1∶2.5),按1.2.4节方法分别固定COX-2,并测定各条件下对塞来昔布的垂钓效率,以确定最优COX-2固定条件。结果如图4b所示,在料液比为1∶2时垂钓效率最高,即当MNPs的质量与COX-2溶液体积为1∶2时,COX-2能够较高效率地被固定。

### 2.3 吸附条件优化

#### 2.3.1 吸附时间

在固定化COX-2缓冲溶液中加入50 μL Tris-HCl缓冲溶液与9 μL塞来昔布溶液,在35 ℃下分别吸附10、30、60、80、90 min,并按1.3.2节条件进行洗脱,将得到的洗脱液分别用HPLC测定其中塞来昔布的质量浓度,计算垂钓效率,结果见图5a。可以看出,垂钓效率随吸附时间逐渐升高,到60 min时趋于平稳,表明此时吸附已达到饱和,故选择60 min作为最优吸附时间。

**图 5:**
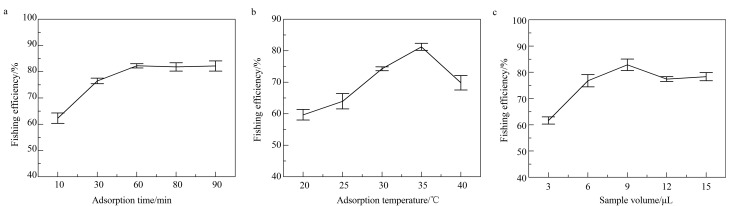
固定化COX-2吸附塞来昔布使用不同(a)吸附时间、(b)吸附温度及(c)样品体积时的垂钓效率(*n*=3)

#### 2.3.2 吸附温度

在固定化COX-2缓冲溶液中加入50 μL Tris-HCl缓冲溶液与9 μL塞来昔布溶液,分别在20、25、30、35、40 ℃下吸附1 h,按1.3.2节方法进行洗脱。将洗脱液分别进行HPLC测定,计算垂钓效率。各结果如图5b所示,垂钓效率随温度升高而增加,在35 ℃时达到最高,而当温度升至40 ℃时,垂钓效率急剧降低,推测温度较高影响了COX-2的活性导致垂钓效率降低。

#### 2.3.3 样品体积

分别在固定化COX-2缓冲溶液中加入3、6、9、12、15 μL塞来昔布溶液并按1.3.2节方法进行吸附和洗脱,收集各洗脱液,由HPLC测定质量浓度,并计算垂钓效率,结果如图5c所示。垂钓效率在样品体积为9 μL时垂钓效率最高,在样品体积增加至12及15 μL时反而有所下降,推测样品量较多可能对固定化COX-2的活性有所影响,故选用9 μL作为样品最终所用体积。

### 2.4 洗脱条件优化

#### 2.4.1 洗脱溶剂

在固定化COX-2缓冲溶液中加入9 μL塞来昔布溶液,按1.3.2节方法进行吸附,然后分别用60%、70%、80%、90%、100%甲醇各400 μL进行洗脱,收集各洗脱液进行HPLC测定,计算垂钓效率。结果见图6a,甲醇比例由60%升至90%,垂钓效率增加但不太明显,而当使用纯甲醇进行洗脱时,垂钓效率明显升高,表明纯甲醇能有效促使COX-2与塞来昔布之间作用键的断裂。

**图 6:**
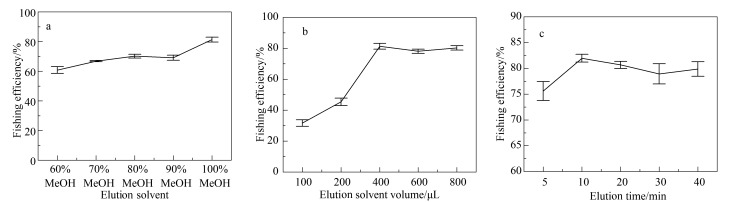
采用不同(a)洗脱溶剂、(b)洗脱溶剂体积及(c)洗脱时间时目标物的垂钓效率(*n*=3)

#### 2.4.2 洗脱溶剂体积

在固定化COX-2缓冲溶液中加入50 μL Tris-HCl缓冲溶液与9 μL塞来昔布溶液,按1.3.2节方法进行吸附,然后分别用100、200、400、600、800 μL甲醇进行洗脱,各洗脱液分别进行HPLC分析,计算垂钓效率,其结果如图6b所示,甲醇体积较低时洗脱的塞来昔布较少,随着甲醇体积的升高,垂钓效率明显升高,在400 μL时达到最高,而在甲醇体积为600及800 μL时垂钓效率无明显变化,表明400 μL甲醇即可将塞来昔布洗脱完全,故确定400 μL为洗脱剂最终所用体积。

#### 2.4.3 洗脱时间

在固定化COX-2缓冲溶液中加入50 μL Tris-HCl缓冲溶液与9 μL塞来昔布溶液,按1.3.2节条件进行吸附,并分别用400 μL甲醇孵育5、10、20、30、40 min,收集洗脱液进行HPLC分析,计算垂钓效率。如图6c所示,洗脱时间在10 min时垂钓效率最高,并在洗脱时间增加时无明显变化。表明在10 min时塞来昔布已基本洗脱完全,故选择10 min作为最优洗脱时间。

### 2.5 方法学考察

#### 2.5.1 线性关系

分别称取一定量的塞来昔布,配制成质量浓度为0.1、0.5、1、2、3、5、9、12、17 mg/mL的溶液,分别进行HPLC分析,以质量浓度为横坐标(*X*),峰面积(*Y*)为纵坐标绘制标准曲线,得到线性回归方程*Y*=129293.41+666962.71*X*, *R*^2^=0.9995,表明在0.1~17 mg/mL范围内具有良好的线性相关性。将0.1 mg/mL的塞来昔布溶液同时进样11次,由检测得到的峰面积计算检出限(LOD)为0.02 mg/mL、定量限(LOQ)为0.07 mg/mL,计算公式为LOD=3SD/*k*, LOQ=10SD/*k*,其中SD为11次进样测得峰面积数值的标准差,*k*为线性回归方程的斜率。

#### 2.5.2 精密度

精密吸取塞来昔布溶液(5 mg/mL),平行制备6份样品,在一天内同时进样并测定含量,计算日内精密度RSD值为2.1%。

吸取塞来昔布标准溶液(5 mg/mL),平行制备6份样品,连续6天进样测定含量,计算其日内精密度RSD值为2.5%。

日内与日间RSD值均小于3%,表明仪器精密度良好。

#### 2.5.3 稳定性

按1.3.2节方法分别加入9 μL塞来昔布溶液得到洗脱液,分别在0、2、4、6、8、10 h时进样,由测得峰面积值计算其质量浓度,RSD值为2.2%,表明样品在10 h内稳定性良好。

#### 2.5.4 重复性

按1.3.2节方法分别加入9 μL塞来昔布溶液平行得到6份洗脱液,分别进行HPLC分析,计算其质量浓度的RSD值为2.5%,表明方法重复性良好。

#### 2.5.5 加标回收率

按1.3.2节方法分别加入质量浓度为3、5、7 mg/mL的塞来昔布溶液制备洗脱液,每个水平均平行制备3份,通过HPLC测定各样品含量,计算得塞来昔布的回收率为85.3%~94.1%, RSD值为0.7%~3.4%,表明该方法具有较好的准确性及适用性。

### 2.6 特异性垂钓验证

以不与COX-2作用的格列吡嗪作为阴性对照、COX-2非特异性抑制剂吲哚美辛以及特异性抑制剂塞来昔布作为阳性对照,对固定化COX-2配体垂钓的特异性、选择性进行验证。

3 种对照品分别称取相同的量加入DMSO溶解,配制成质量浓度均为2 mg/mL的混合标准溶液。按1.3.2节方法向固定化COX-2缓冲溶液中加入9 μL混合标准溶液进行吸附与洗脱,收集洗脱液与混合标准溶液分别进行HPLC分析。

垂钓结果如图7所示,a为混合标准溶液色谱图,其中峰1为格列吡嗪,峰2为吲哚美辛,峰3为塞来昔布;图7b为垂钓后洗脱液色谱图,可以看到只有塞来昔布的峰,同时在垂钓剩余液(图7c)中可以看到格列吡嗪、吲哚美辛的峰面积并无明显地变化,而塞来昔布峰面积明显变小,即塞来昔布基本被垂钓出。以上表明固定化COX-2具有良好的垂钓特异性,且能垂钓出与COX-2作用强的成分。

**图 7:**
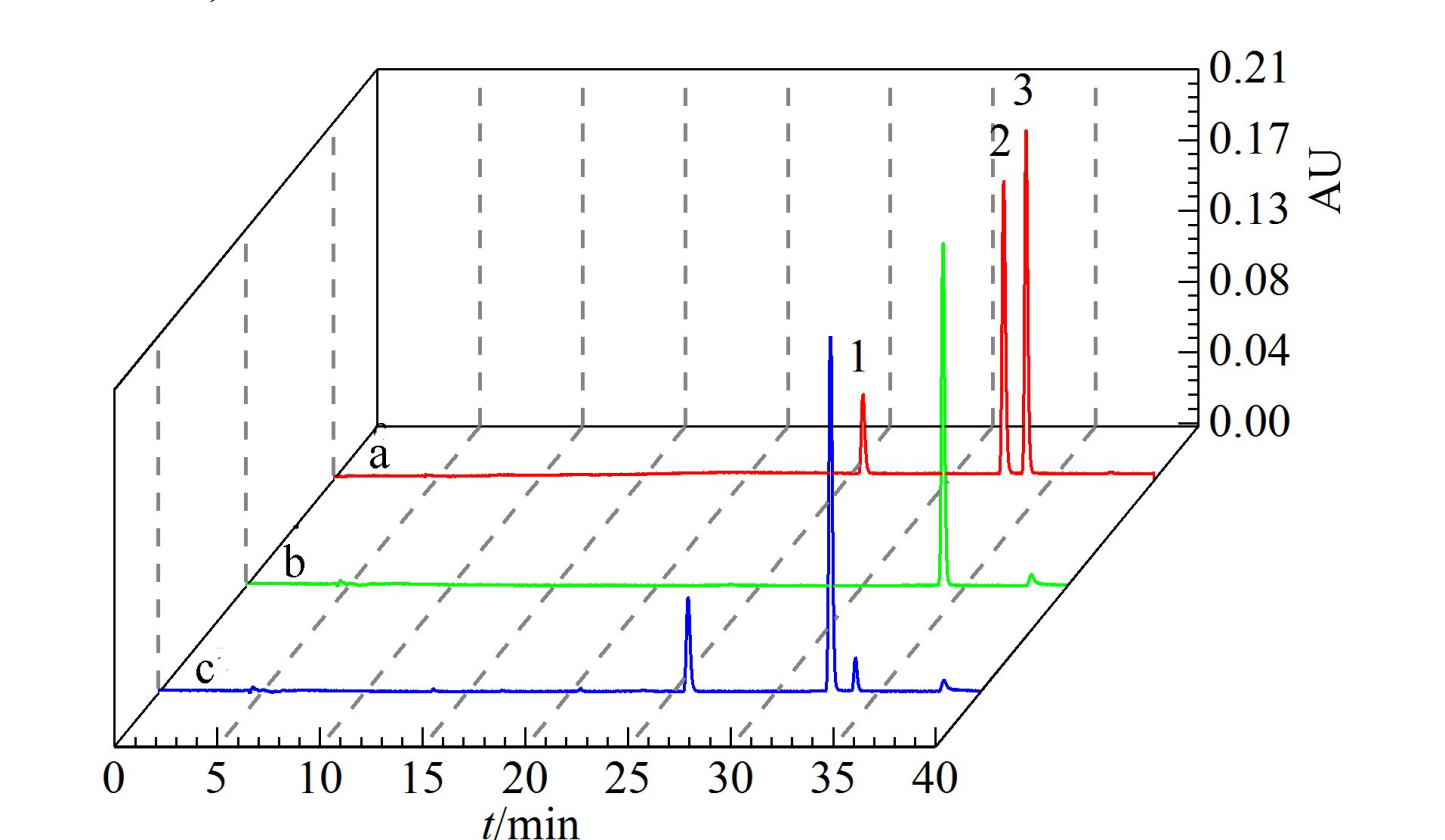
(a)混合标准溶液、(b)固定化COX-2垂钓液及(c)垂钓后剩余液的色谱图

### 2.7 垂钓成分鉴定

通过比较垂钓洗脱液与垂钓前三七液总皂苷提取液的液相色谱-质谱总离子流图,最终确定从三七叶中垂钓到了13种皂苷成分,结合各峰的分子质量、碎片离子峰等信息,通过与标准品比对,最终确定13种化合物的名称及苷元结构见图8及表2;13种皂苷均为20(*S*)-原人参二醇型三萜皂苷,分别连接2~5个单糖。三七叶提取液及垂钓液的总离子流色谱图见图9。

**图 8:**
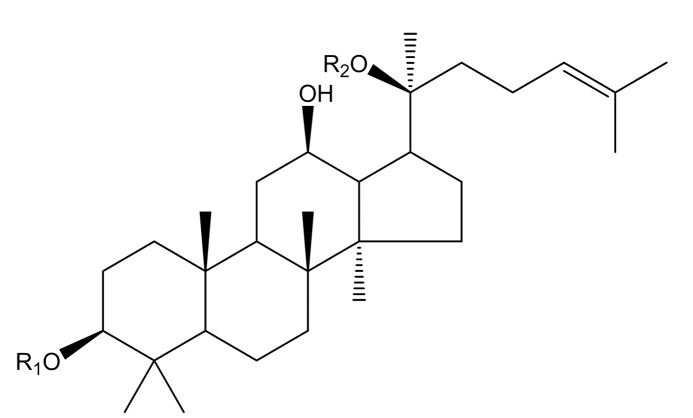
13种皂苷的苷元结构图

**表 2:** 垂钓液中13种皂苷成分的名称及结构信息

No.	t/min	Experimental m/z ([M-H]^-^)	Theoretical m/z ([M-H]^-^)		Error/ 10^-6^		Formula		Compound		R_1_ in Fig. 8		R_2_ in Fig. 8
1	17.03	1239.6421	1239.6374	3.8		C_59_H_100_O_27_		notoginsenoside Fa		-Glc(2-1)Glc(2-1)Xyl		-Glc(6-1)Glc	
2	18.13	1209.6316	1209.6268	4.0		C_58_H_98_O_26_		notoginsenoside FP_2_		-Glc(2-1)Glc(2-1)Xyl		-Glc(6-1)Ara(f)	
3	18.37	1107.6001	1107.5951	4.5		C_54_H_92_O_23_		ginsenoside Rb_1_		-Glc(2-1)Glc		-Glc(6-1)Glc	
4	19.49	1077.5889	1077.5845	4.1		C_53_H_90_O_22_		ginsenoside Rc		-Glc(2-1)Glc		-Glc(6-1)Ara(f)	
5	19.83	1209.6323	1209.6268	4.5		C_58_H_98_O_26_		notoginsenoside Fc		-Glc(2-1)Glc(2-1)Xyl		-Glc(6-1)Xyl	
6	20.76	1077.5886	1077.5845	3.8		C_53_H_90_O_22_		ginsenoside Rb_2_		-Glc(2-1)Glc		-Glc(6-1)Ara(p)	
7	21.24	1077.5886	1077.5845	3.8		C_53_H_90_O_22_		ginsenoside Rb_3_		-Glc(2-1)Glc		-Glc(6-1)Xyl	
8	23.22	945.5454	945.5423	3.3		C_48_H_82_O_18_		ginsenoside Rd		-Glc(2-1)Glc		-Glc	
9	25.51	945.5447	945.5423	2.5		C_48_H_82_O_18_		gypenoside ⅩⅦ		-Glc		-Glc(6-1)Glc	
10	27.09	915.5333	915.5317	1.7		C_47_H_80_O_17_		notoginsenoside Fe		-Glc		-Glc(6-1)Ara(f)	
11	28.43	915.5352	915.5317	3.8		C_47_H_80_O_17_		vina-ginsenoside R_18_		-Glc(2-1)Glc		-Xyl	
12	28.94	915.5338	915.5317	2.3		C_47_H_80_O_17_		gypenoside Ⅸ		-Glc		-Glc(6-1)Xyl	
13	35.20	783.4907	783.4894	1.7		C_42_H_72_O_13_		ginsenoside F_2_		-Glc		-Glc	

**图 9:**
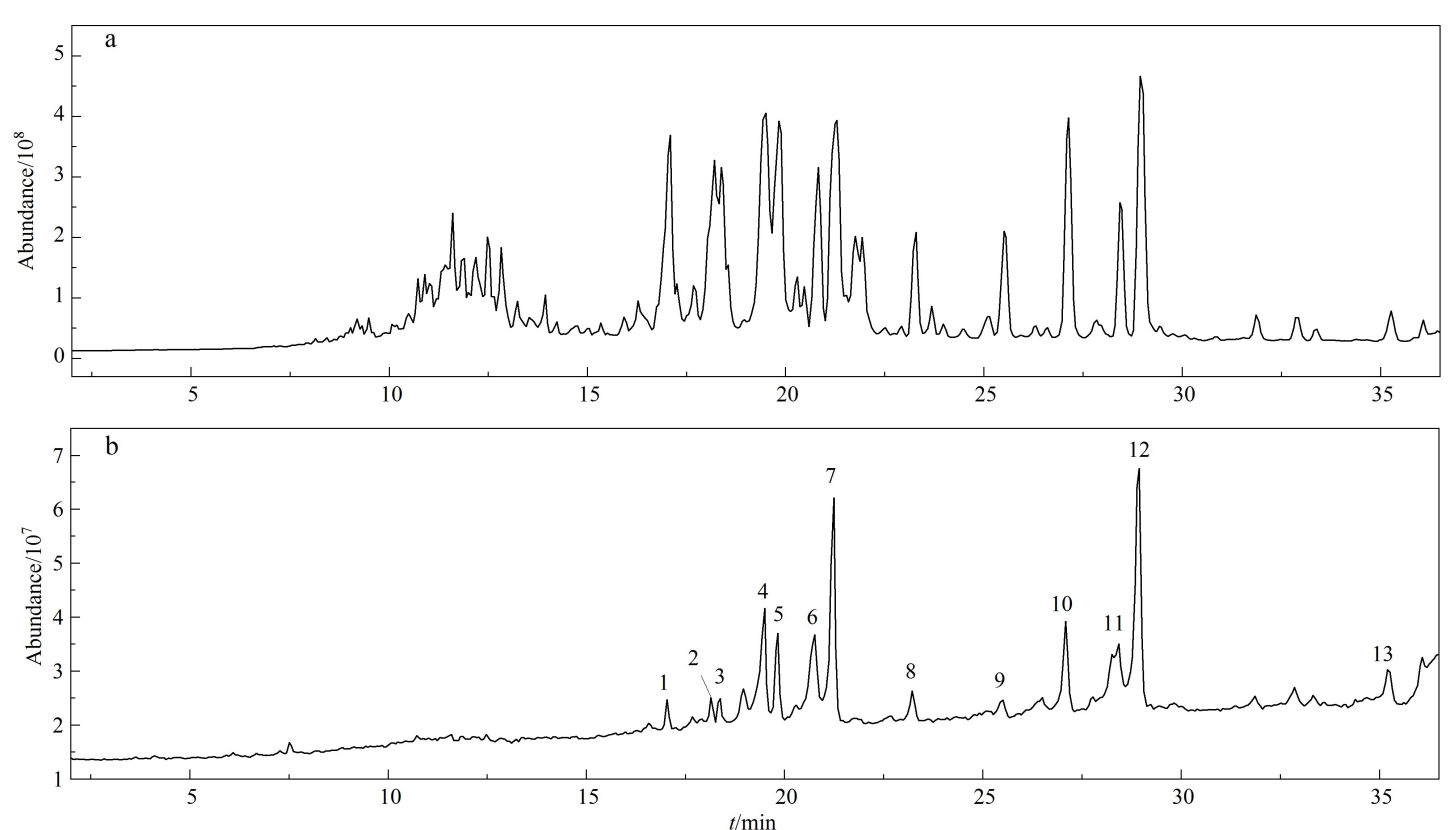
(a)三七叶提取液及(b)垂钓液的总离子流色谱图

## 3 结论

本研究合成了一种复合磁性纳米材料,结合固定化金属离子亲和技术将COX-2固定在MNPs上有效保持了酶固定后的活性,成功用于三七叶中COX-2抑制剂的筛选,从中垂钓得到了13种皂苷成分,有望成为潜在的COX-2抑制剂,不仅为开发新型安全有效的COX-2抑制剂提供了有力地支持,也为抗炎药物的开发及合成提供了新的思路,同时也为三七叶的高效资源化利用提供了理论借鉴。相比于传统的筛选方法,本方法简便快速、特异性强、酶活性高,能够实现对特定目标物的捕获与富集,但筛选的皂苷成分与COX-2的作用位点尚不明确,各成分对COX-2的抑制作用及抗炎活性也需要后续的药理研究进一步探索。
